# Supplementary material for: Splice-Junction-Based Mapping of Alternative Isoforms in the Human Proteome
Source: Cell Rep. Author manuscript; Available in PMC 2020 Jan 15. (PMC6961840; doi:10.1016/j.celrep.2019.11.026)
Supplement: 2 [file NIHMS1546469-supplement-2.zip › DF1/PXD010154/Ovary/Ovary_10_SRSF11_VTAQPDVLEVQAEYITAGPGPSGGPGGGG.pdf]

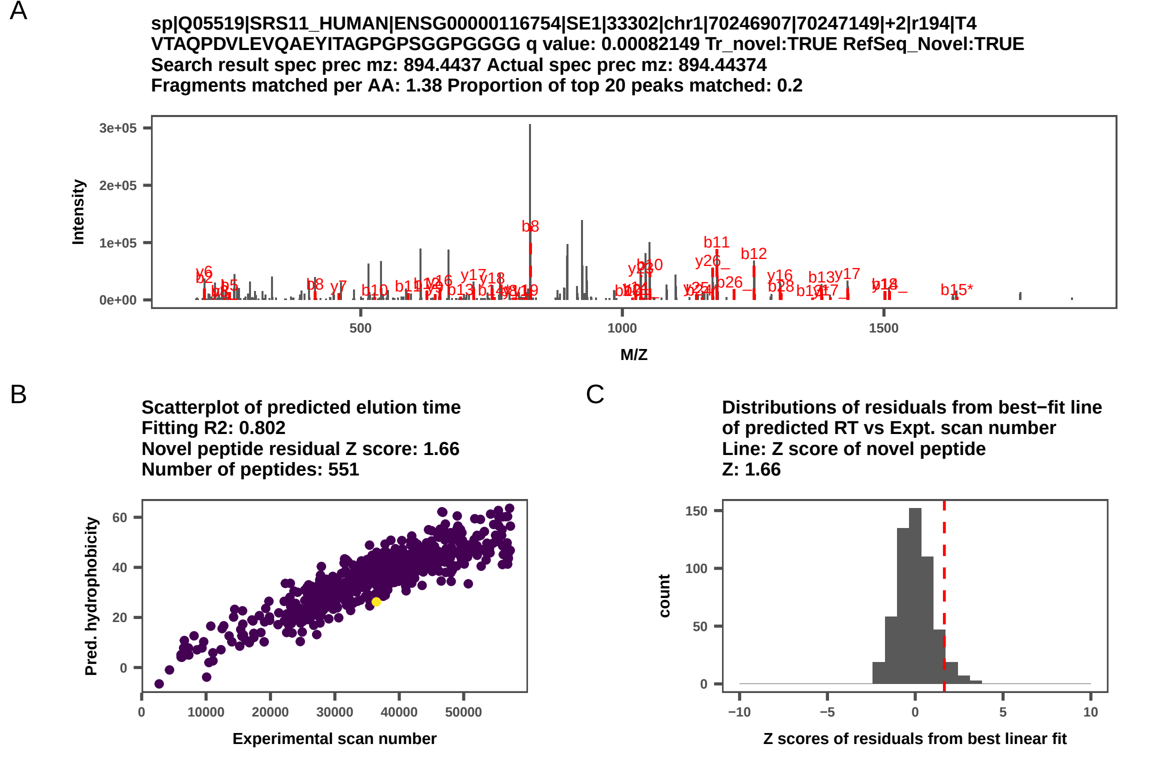
A
sp|Q05519|SRS11_HUMAN|ENSG00000116754|SE1|33302|chr1|70246907|70247149|+2|r194|T4
VTAQPDVLEVQAEYITAGPGPSGGPGGGG q value: 0.00082149 Tr_novel:TRUE RefSeq_Novel:TRUE
Search result spec prec mz: 894.4437 Actual spec prec mz: 894.44374
Fragments matched per AA: 1.38 Proportion of top 20 peaks matched: 0.2
Intensity
3e+05
2e+05
1e+05
0e+00
500
1000
1500
M/Z
b8
b11
b12
y26
b26
y16
b28
y17
b15*
y6
b5
y7
b10
y18
B
Scatterplot of predicted elution time
Fitting R2: 0.802
Novel peptide residual Z score: 1.66
Number of peptides: 551
Pred. hydrophobicity
60
40
20
0
0
10000
20000
30000
40000
50000
Experimental scan number
C
Distributions of residuals from best−fit line
of predicted RT vs Expt. scan number
Line: Z score of novel peptide
Z: 1.66
count
150
100
50
0
−10
−5
0
5
10
Z scores of residuals from best linear fit
